# Admission levels of asymmetric and symmetric dimethylarginine predict long-term outcome in patients with community-acquired pneumonia

**DOI:** 10.1186/s12931-017-0502-4

**Published:** 2017-01-23

**Authors:** Alaadin Vögeli, Manuel Ottiger, Marc A. Meier, Christian Steuer, Luca Bernasconi, Prasad Kulkarni, Andreas Huber, Mirjam Christ-Crain, Christoph Henzen, Claus Hoess, Robert Thomann, Werner Zimmerli, Beat Mueller, Philipp Schuetz

**Affiliations:** 1 Kantonsspital Aarau, University Department of Internal Medicine, Tellstrasse, CH-5001 Aarau, Switzerland; 2 Institute of Laboratory Medicine, Kantonsspital Aarau, Aarau, Switzerland; 3 Asclepius Medical Communications LLC, Ridgewood, NJ USA; 4 Endocrinology, Diabetology, and Metabolism, University Hospital Basel, Basel, Switzerland; 5 Department of Internal Medicine, Kantonsspital Luzern, Luzern, Switzerland; 6 Department of Internal Medicine, Kantonsspital Münsterlingen, Münsterlingen, Switzerland; 7 Department of Internal Medicine, Bürgerspital Solothurn, Solothurn, Switzerland; 8 Kantonsspital Baselland, University Department of Internal Medicine, Liestal, Switzerland

**Keywords:** Asymmetric dimethylarginine (ADMA), Community-acquired pneumonia (CAP), Biomarker, L-arginine, Mortality prediction, Symmetric dimethylarginine (SDMA)

## Abstract

**Background:**

During infection, there is an activation of the L-arginine-nitric-oxide pathway, with a shift from nitric oxide synthesis to a degradation of L-arginine to its metabolites, asymmetric and symmetric dimethylarginine (ADMA and SDMA). However, the prognostic implications for short-term or long-term survival remains unclear. We investigated the association of L-arginine, ADMA, and SDMA with adverse clinical outcomes in a well-defined cohort of patients with community-acquired pneumonia (CAP).

**Methods:**

We measured L-arginine, ADMA, and SDMA in 268 CAP patients from a Swiss multicenter trial by mass spectrometry and used Cox regression models to investigate associations between blood marker levels and disease severity as well as mortality over a period of 6 years.

**Results:**

Six-year mortality was 44.8%. Admission levels of ADMA and SDMA (μmol/L) were correlated with CAP severity as assessed by the pneumonia severity index (*r* = 0.32, *p* < 0.001 and *r* = 0.56, *p* < 0.001 for ADMA and SDMA, respectively) and higher in 6-year non-survivors versus survivors (median 0.62 vs. 0.48; *p* < 0.001 and 1.01 vs. 0.85; *p* < 0.001 for ADMA and SDMA, respectively). Both ADMA and SDMA were significantly associated with long-term mortality (hazard ratios [HR] 4.44 [95% confidence intervals (CI) 1.84 to 10.74] and 2.81 [95% CI 1.45 to 5.48], respectively). The effects were no longer significant after multivariate adjustment for age and comorbidities. No association of L-arginine with severity and outcome was found.

**Conclusions:**

Both ADMA and SDMA show a severity-dependent increase in patients with CAP and are strongly associated with mortality. This association is mainly explained by age and comorbidities.

**Trial registration:**

ISRCTN95122877. Registered 31 July 2006.

## Background

**Fig. 1:**
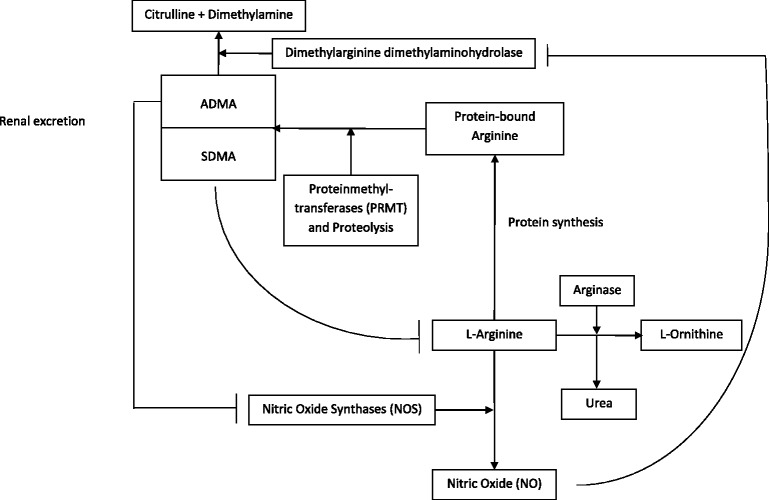
Metabolism of nitric oxide. Nitric oxide (*NO*) is synthesized from L-arginine. This reaction is catalyzed by nitric oxide synthases (*NOS*). Protein synthesis incorporates L-arginine into proteins. Protein-bound L-arginine is methylated by protein methyltransferases (*PRMT*) and lysed to the metabolites asymmetric and symmetric dimethylarginine (*ADMA*, *SDMA*). ADMA is mainly metabolized to citrulline and dimethylamine via dimethylarginine dimethylaminohydrolase (*DDAH*). A small fraction of ADMA and almost the entire amount of SDMA are renally eliminated. ADMA inhibits the NOS competitively and SDMA interferes with L-arginine transport. Arginase reduces the bioavailability of L-arginine by degrading it to L-ornithine and urea. Nitric oxide has a negative feedback mechanism to DDAH, which leads to less metabolism of ADMA

Nitric oxide (NO), which is synthesized from L-arginine by nitric oxide synthases (NOS), is a vaso- and bronchodilator [1]. Its deficiency results in airway hyperreactivity [2] and endothelial dysfunction [3]. In addition, nitric oxide inhibits platelet adhesion and is considered to have a role in nonspecific immunity [4]. Arginase, which converts L-arginine into L-ornithine and urea, reduces the bioavailability of L-Arginine for NOS and thereby inhibits the L-Arginine-Nitric oxide pathway [5]. L-arginine residues are methylated by protein methyltransferases (PRMT). Asymmetric and symmetric dimethylarginine (ADMA and SDMA) are derived from the proteolysis of these methylated arginines on various proteins (Fig. 1) [6, 7]. While ADMA acts as a competitive inhibitor of the NOS, SDMA is a competitor of arginine transport but does not interfere with NOS [8]. Thus, an elevated serum ADMA level results in lowered NO levels, which leads to vasoconstriction, augmented thrombocyte aggregation, and cell adhesion to the endothelium and promotes proliferation of vascular muscle cells [9]. ADMA is metabolized to citrulline and dimethylamine by the enzyme dimethylarginine dimethylaminohydrolase (DDAH) and a small fraction is renally excreted whereas SDMA is almost entirely eliminated by the kidneys. There is also a negative feedback loop of NO, with high NO concentrations leading to the inhibition of DDAH [10].

Circulating levels of ADMA are elevated in multiple diseases with endothelial dysfunction (e.g., hypertension, hyperlipidemia, diabetes mellitus, atherosclerosis, and renal failure) [3, 6, 11–14], in diseases of the respiratory system (e.g., asthma and COPD) [2, 15], and in multiple organ failure in sepsis [16]. Its isomer, SDMA, is also associated with a higher prevalence of cardiovascular risk factors as well as all-cause and cardiovascular mortality [10, 17]. Increased plasma concentrations of ADMA and SDMA are independent prognostic factors for short-term and long-term mortality in critically ill patients [18, 19]. In contrast, high levels of serum SDMA, but not of ADMA, seem to be an independent prognostic biomarker of all-cause mortality in the general population [17]. Both, ADMA and SDMA are increased in elder people [20]. In addition, a lowered ratio of arginine to ADMA is a sensitive risk marker for atherosclerosis, cardiovascular disease, and mortality in patients with shock, being even more sensitive than ADMA alone [21, 22].

Community-acquired pneumonia (CAP) is to date one of the leading causes of death due to an infection [23]. CAP is also associated with an increased risk of cardiovascular events and, thus, is an independent cardiovascular risk factor [24–26]. The aim of our study was to evaluate the role of L-arginine, ADMA, and SDMA in the pathophysiology of CAP. We also wanted to determine whether the ratio of arginine to ADMA correlates with disease severity and mortality in CAP patients over a follow-up period of 6 years.

## Methods

### Study design

A total of 268 patients with a definite diagnosis of CAP and available blood samples from a previous Swiss multicenter trial (the ProHOSP trial) [27, 28] were included in this investigation. The initial trial included 925 (68%) CAP patients with an all-cause mortality of 45% who were followed up over a period of 6 years [29].

The initial trial was a noninferiority randomized controlled trial evaluating the economical use of antibiotics at six Swiss secondary and tertiary care centers between October 2006 and March 2008 [27]. The primary goal of the study was to verify the efficacy and safety of using serum procalcitonin (PCT) levels to guide the initiation and duration of antibiotic therapy in patients with lower respiratory tract infections [27, 28]. Patients ≥18 years of age presenting from the community or a nursing home to the emergency department (ED), were included if they met at least one of the following criteria: cough, dyspnea, pleural pain, sputum production, or tachypnea and one sign of an infection (core body temperature >38 °C, shivering, white blood cell count >10 or <4x10^9^ cells/L), or one clinical finding upon auscultation (rales or crepitation). An infiltrate was radiologically confirmed in all patients with CAP. Exclusion criteria included language restriction or dementia that precluded patients from giving written informed consent, intravenous drug use, a terminal condition, or hospital-acquired pneumonia (HAP). Participants were accepted into the study if they had received short-term antibiotic pretreatment or corticosteroids, but were excluded if they had obtained long-term antibiotic pretreatment or suffered from severe immunosuppression at ED presentation.

### Analysis of blood biomarkers

Blood samples collected from each patient and frozen in aliquots at ED admission were used for measuring the different biomarkers. Baseline arginine, ADMA, and SDMA levels were measured in blood samples of all 268 CAP patients included in the study, with 109 of them also having blood specimens available at day 7 of hospitalization for these measurements. If any of above biomarkers had a value of 0, it was considered to be a measuring error and excluded from the analysis. Due to technical errors or insufficient volume of blood specimens during biomarker measurement, not all patients had a complete set of markers. The final analysis included 268 patients of which 268 (100%) had complete markers of arginine, while 233 (87%) and 242 (90%) had ADMA and SDMA markers available, respectively.

Liquid chromatography tandem-mass spectrometry (LC-MS/MS) analysis was performed using an Ultimate 3000 UHPLC (Thermo Fisher, San Jose, USA) system coupled to an ABSciex 5500 quadrupole mass spectrometer (ABSciex, Darmstadt, Germany) and the *AbsoluteIDQ* p180 Kit (BIOCRATES Life Sciences AG, Innsbruck, Austria) [30–32]. Sample preparation and measurements were performed as described in the AbsoluteIDQ p180 user’s manual. Chromatographic separation was performed on a Thermo Syncronis aQ 50x2.1 mm 1.7 μm column. The prepared samples were analyzed using multiple reaction monitoring (MRM). Selected metabolites were quantified by reference to appropriate internal standards. All concentrations were reported in μM.

### Main outcome measurements

The primary endpoint was all-cause mortality after a follow-up period of 6 years. Secondary endpoints were mortality at day 30 and 1-year, ICU admissions, and clinical findings (pneumonia severity index [PSI] and quick Sepsis Related Organ Failure Assessment [qSOFA] score) [33]. Vital status was ascertained through structured phone interviews by trained medical students at days 30, 180, and 540, and at 6 years [29, 34]. If patients or their household members could not be reached, the treating general practitioners were contacted.

### Statistical analyses

All statistical analyses were performed using STATA 12.1 (Stata Corp, College Station, TX, USA). Statistical significance was considered at a *p* value <0.05. Categorical variables are expressed as percentages (numbers) and continuous variables as medians (interquartile range [IQR]) unless stated otherwise. The distribution of ADMA, SDMA, and L-arginine (just referred to as biomarkers henceforth) were skewed. The distribution of the biomarkers approximated a normal distribution after logarithmic transformation at a base of 10. Chi-square (Wald) tests were used for frequency comparisons, and nonparametric tests (Mann-Whitney U, Kruskal-Wallis, and Spearman’s rank correlation) were executed for comparisons of two or more groups. The significance levels were subjected to Bonferroni adjustment. To study the association of comorbidities with initial biomarker levels, we used univariate and multivariate linear regression models. To investigate associations between biomarker levels at baseline and all-cause mortality, we utilized univariate and multivariate Cox regression models. These associations are reported as hazard ratios (HRs) with 95% confidence intervals (CIs) and significance levels for the chi-square (Wald) test. Odds ratios were calculated and reported with 95% confidence intervals (CIs). Area under the receiver operating characteristics curves (AUCs) with 95% CIs are presented to illustrate discrimination and predictive power. Mortality based on biomarker quartiles (highest versus lower three) has been illustrated through the use of Kaplan-Meier curves. All analyses were performed with the ratio of arginine to ADMA as well.

## Results

### Patient characteristics

Baseline characteristics of the entire cohort (*N* = 268) as well as for patients stratified by 6-year survival status are presented in Table 1. The median age of the entire cohort was 72 years and 40.7% were female. There was a high burden of comorbidities, with 18.3% having a history of coronary artery disease (CAD), 23.9% having chonic renal failure (CRF), and 26.1% having chronic obstructive pulmonary disease (COPD).

**Table 1:** Baseline characteristics: overall and by 6-year vital status

Factor		Total	Survivor	Non-Survivor	*p* value
*N*		268	148	120	
Demographic characteristics					
Age, median (IQR)		72 (57, 82)	63 (43, 75)	80 (71, 86)	**<0.001**
Gender, *n* (%)	Male	159 (59.3%)	78 (52.7%)	81 (67.5%)	**0.014**
	Female	109 (40.7%)	70 (47.3%)	39 (32.5%)	
Smoking status					
Current Smoker, *n* (%)	Non-smoker	193 (72.0%)	96 (64.9%)	97 (80.8%)	**0.004**
	Smoker	75 (28.0%)	52 (35.1%)	23 (19.2%)	
Ever-Smoker, *n* (%)	Never-smoker	169 (63.1%)	86 (58.1%)	83 (69.2%)	0.062
	Ever-smoker	99 (36.9%)	62 (41.9%)	37 (30.8%)	
Comorbidites^a^					
Coronary artery disease		49 (18.3%)	12 (8.1%)	37 (30.8%)	**<0.001**
Congestive heart failure		33 (12.3%)	6 (4.1%)	27 (22.5%)	**<0.001**
Cerebrovascular disease		26 (9.7%)	7 (4.7%)	19 (15.8%)	**0.002**
Peripheral arterial occlusive disease		15 (5.6%)	4 (2.7%)	11 (9.2%)	**0.022**
Chronic renal failure		64 (23.9%)	19 (12.8%)	45 (37.5%)	**<0.001**
Diabetes mellitus		41 (15.3%)	17 (11.5%)	24 (20.0%)	0.054
Tumor		32 (11.9%)	9 (6.1%)	23 (19.2%)	**0.001**
Chronic obstructive pulmonary disease		70 (26.1%)	32 (21.6%)	38 (31.7%)	0.063
Vital signs					
Fever, *n* (%)		174 (65.2%)	111 (75.0%)	63 (52.9%)	**<0.001**
Chills, *n* (%)		85 (36.0%)	59 (43.7%)	26 (25.7%)	**0.004**
Pulse, median (IQR)		94 (82, 106)	94 (83, 108)	94 (80, 104)	0.21
Temperature, median (IQR)		38.0 (37.1, 38.8)	38.2 (37.3, 39.0)	37.8 (37.0, 38.8)	0.084
Systolic BP in mmHg, median (IQR)		130 (118, 146)	132 (120, 146)	130 (110, 148)	0.12
Severity scores					
PSI	1, 2	72 (26.9%)	65 (43.9%)	7 (5.9%)	**<0.001**
	3	55 (20.5%)	36 (24.3%)	19 (15.8%)	
	4, 5	141 (52.7%)	47 (31.7%)	94 (78.4%)	
qSOFA score	0	116 (43.3%)	78 (52.7%)	38 (31.7%)	**<0.001**
	1	127 (47.4%)	67 (45.3%)	60 (50.0%)	
	2, 3	25 (9.4%)	3 (2.0%)	22 (18.3%)	
Metabolite concentrations (μM)					
Arginine		42.8 (28.6, 63.2)	43.3 (28.2, 64.3)	41.3 (29.6, 62.2)	0.97
ADMA		0.56 (0.42, 0.73)	0.48 (0.38, 0.67)	0.62 (0.47, 0.83)	**<0.001**
SDMA		0.85 (0.56, 1.15)	0.78 (0.52, 1.00)	1.01 (0.63, 1.38)	**<0.001**
Arginine/ADMA Ratio		76.18 (54.08, 113.50)	85.11 (62.39, 116.58)	66.52 (48.51, 106.77)	**0.004**

Bolded *p* values are statistically significant at *p* < 0.05

*Abbreviations*: *ADMA* asymmetric dimethylarginine, *BP* blood pressure, *PSI* pneumonia severity index, *qSOFA* quick sepsis related organ failure assessment (sepsis risk score), *SDMA* symmetric dimethylarginine

^a^Comorbidities were identified based on medical records, patient report, or both

### Association between the investigated biomarkers (L-arginine, ADMA, SDMA) and mortality

**Fig. 2:**
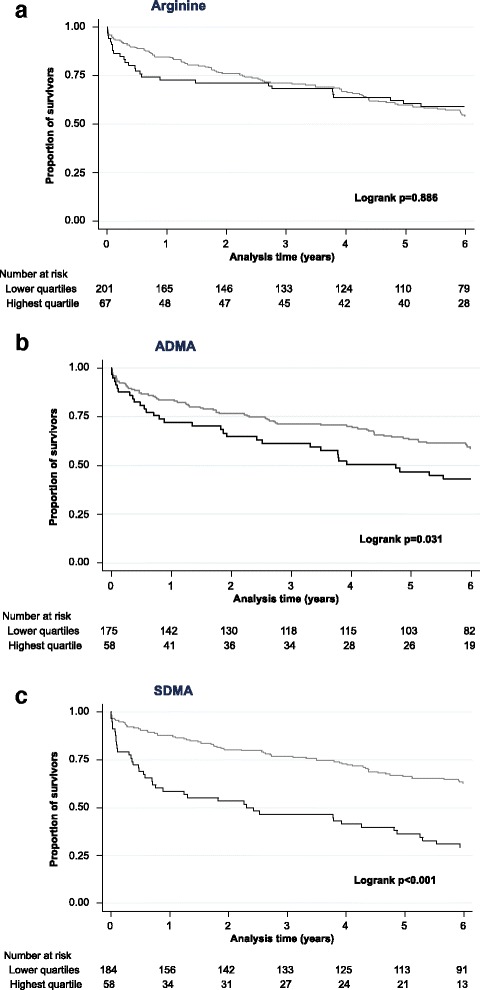
Kaplan-Meier survival estimates for arginine (**a**) asymmetric dimethylarginine (ADMA) (**b**) and symmetric dimethylarginine (SDMA) (**c**). *Grey* represents the lower three quartiles and *black* the highest quartile

Mortality at day 30 was 5.2% (*n* = 14), increasing to 17.9% (*n* = 48) after 1 year and 44.8% (*n* = 120) after 6 years of follow up. Figure 2 shows Kaplan-Meier curves for 6-year all-cause mortality based on the highest quartile compared to lower quartiles of arginine, ADMA and SDMA.

Baseline levels of ADMA and SDMA were increased in 6-year non-survivors compared with survivors (ADMA median, 0.62 μmol/L [IQR 0.47–0.83] vs. 0.48 μmol/L [IQR 0.38–0.67]; *p* <0.001 and SDMA median, 1.01 μmol/L [IQR 0.63–1.38] vs. 0.85 μmol/L [IQR 0.52–1.00]; *p* <0.001). There were no significant differences in arginine levels between non-survivors and survivors (median, 41.3 μmol/L [IQR 29.6–62.2] vs. 43.3 μmol/L [IQR 28.2–64.3]; *p = 0.97*) (see Table 1).

In Cox regression analyses, while ADMA, SDMA, and their ratios were found to be associated with long-term mortality, only SDMA was related to short-term mortality. This association did not remain robust following adjustment for different comorbidities. Further analyses revealed an AUC of SDMA of 0.71 (95% CI 0.56 to 0.87) for short- and mid-term mortality and 0.64 (95% CI 0.57 to 0.71) for long-term mortality at 6 years. The AUCs for ADMA did not reach these levels (Table 2).

**Table 2:** Association of initial biomarker levels with all-cause mortality at different time points

	All-cause mortality time point		
Entire Cohort (*N* = 268)	30 day	1 year	6 years
Cox regression models	HR (95% CI); *p* value		
Arginine			
Univariate	1.04 (0.17 to 6.31); *p* = 0.968	2.19 (0.71 to 6.79); *p* = 0.175	1.10 (0.56 to 2.15), *p* = 0.791
Multivariate^a^	1.08 (0.14 to 8.45); *p* = 0.942	2.53 (0.74 to 8.74); *p* = 0.141	1.12 (0.52 to 2.41); *p* = 0.764
AUC	0.52 (0.33 to 0.71)	0.56 (0.46 to 0.66)	0.50 (0.43 to 0.57)
ADMA			
Univariate	10.47 (0.72 to 152.55); *p* = 0.086	7.22 (1.81 to 28.77)**;** ***p*** **= 0.005**	4.44 (1.84 to 10.74)**;** ***p*** **= 0.001**
Multivariate^a^	5.79 (0.29 to 116.72); *p* = 0.252	3.33 (0.72 to 15.39); *p* = 0.124	1.91 (0.73 to 5.00); *p* = 0.188
AUC	0.67 (0.52 to 0.82)	0.67 (0.58 to 0.75)	0.65 (0.58 to 0.72)
SDMA			
Univariate	15.79 (2.23 to 112.02)**;** ***p*** **= 0.006**	5.62 (1.92 to 16.46)**;** ***p*** **= 0.002**	2.81 (1.45 to 5.48)**;** ***p*** **= 0.002**
Multivariate^a^	2.78 (0.17 to 44.74); *p* = 0.471	1.22 (0.39 to 3.86); *p* = 0.736	0.82 (0.44 to 1.52); *p* = 0.523
AUC	0.71 (0.56 to 0.87)	0.70 (0.61 to 0.8)	0.64 (0.57 to 0.71)
Arginine/ADMA Ratio			
Univariate	0.36 (0.04 to 3.78); *p* = 0.398	0.44 (0.13 to 1.54); *p* = 0.198	0.33 (0.15 to 0.74)**;** ***p*** **= 0.007**
Multivariate^a^	0.93 (0.12 to 7.31); *p* = 0.944	1.25 (0.33 to 4.68); *p* = 0.741	0.84 (0.36 to 1.93); *p* = 0.675
AUC	0.44 (0.25 to 0.63)	0.43 (0.32 to 0.53)	0.39 (0.32 to 0.46)

Data for univariate and multivariate Cox regression models are given as hazard ratio (95% CI); *p* value

Data regarding discrimination (prognostic accuracy) are given as area under the receiver operating characteristic curve (95% CI)

*P* values in bold type are statistically significant at *p* < 0.05

*Abbreviations*: *ADMA* Asymmetric Dimethylarginine, *AUC* area under the receiver operating characteristics curve, *CI* confidence interval, *HR* hazard ratio, *SDMA* Symmetric Dimethylarginine

^a^Multivariate model: Admission concentration of logarithmic biomarkers adjusted for comorbidities (coronary artery disease, congestive heart failure, cerebrovascular disease, peripheral artery occlusive disease, diabetes mellitus, chronic renal failure, neoplastic disease, and chronic obstructive pulmonary disease) and age

### Association between biomarkers and severity

In Spearman’s rank correlation analyses with Bonferroni adjustment, we found significant associations of ADMA and SDMA with the Pneumonia Severity Index (*ρ* = 0.31, *p* <0.001 and *ρ* = 0.5307, *p* <0.001). In contrast, there was no association between all the investigated biomarkers and the qSOFA score.

### Association of biomarkers with comorbidities

We developed linear regression models investigating predictors for increased biomarker levels. Univariate analysis showed significant correlation of SDMA and ADMA to numerous comorbidities and patient characteristics (Table 3). The multivariate models revealed that higher levels of SDMA are seen in patients with chronic renal failure, COPD, and in older patients. Levels of SDMA therefore showed a better correlation to comorbidities than ADMA, which was associated only with age and neoplastic disease. Levels of arginine did not show any correlation whatsoever (Table 4).

**Table 3:** Univariate association of initial biomarker levels with demographic characteristics and comorbidities

	Arginine		ADMA		SDMA		Arginine/ADMA ratio	
	Coefficient (95% CI)	*p* value	Coefficient (95% CI)	*p* value	Coefficient (95% CI)	*p* value	Coefficient (95% CI)	*p* value
Patient characteristics								
Age in decades	0.01 (−0.01 to 0.03)	0.246	0.03 (0.01 to 0.04)	**<0.001**	0.07 (0.05 to 0.10)	**<0.001**	−0.07 (−0.10 to −0.04)	**<0.001**
Female	−0.02 (−0.09 to 0.05)	0.546	0.001 (−0.05 to 0.05)	0.958	−0.01 (−0.11 to 0.09)	0.856	−0.01 (−0.12 to 0.10)	0.830
BMI	−0.01 (−0.01 to 0.001)	0.065	0.002 (−0.003 to 0.01)	0.361	0.0001 (−0.01 to 0.01)	0.971	−0.01 (−0.02 to 0.01)	0.297
Systolic blood pressure in 10 mmHg	0.01 (0.0004 to 0.03)	0.143	−0.01 (−0.02 to 0.005)	0.260	−0.02 (−0.04 to −0.004)	**0.020**	0.03 (0.01 to 0.06)	**0.005**
Comorbidities								
Coronary artery disease	−0.08 (−0.17 to 0.01)	0.076	−0.01 (−0.08 to 0.05)	0.719	0.15 (0.03 to 0.27)	**0.014**	−0.26 (−0.39 to −0.13)	**<0.001**
Congestive heart failure	−0.01 (−0.12 to 0.10)	0.851	0.05 (−0.03 to 0.12)	0.204	0.17 (0.03 to 0.31)	**0.017**	−0.22 (−0.38 to −0.06)	**0.007**
Cerebrovascular disease	0.10 (−0.02 to 0.22)	0.091	0.05 (−0.04 to 0.13)	0.272	0.15 (−0.004 to 0.31)	0.057	−0.08 (−0.26 to 0.10)	0.387
PAOD	0.05 (−0.11 to 0.20)	0.546	−0.01 (−0.11 to 0.10)	0.901	0.15 (−0.05 to 0.35)	0.133	−0.13 (−0.35 to 0.10)	0.274
Chronic renal failure	0.02 (−0.06 to 0.11)	0.603	0.07 (0.01 to 0.12)	**0.027**	0.32 (0.22 to 0.43)	**<0.001**	−0.32 (−0.44 to −0.20)	**<0.001**
Diabetes mellitus	0.05 (−0.05 to 0.14)	0.362	0.06 (−0.01 to 0.13)	0.111	0.15 (0.02 to 0.28)	**0.021**	−0.14 (−0.29 to 0.01)	**0.064**
Neoplastic disease	0.03 (−0.08 to 0.14)	0.578	0.10 (0.03 to 0.17)	**0.008**	0.17 (0.02 to 0.31)	**0.022**	−0.16 (−0.32 to 0.01)	0.060
COPD	−0.01 (−0.09 to 0.07)	0.790	−0.03 (−0.08 to 0.03)	0.361	0.17 (0.06 to 0.27)	**0.002**	−0.18 (−0.30 to −0.06)	**0.004**

Data for univariate linear regression analyses are given as coefficient (95% CI)

Bolded *p* values are statistically significant at *p* < 0.05

*Abbreviations*: *ADMA* asymmetric dimethylarginine, *BMI* body mass index, *CI* confidence interval, *COPD* chronic obstructive pulmonary disease, *PAOD* peripheral artery occlusive disease, *SDMA* symmetric dimethylarginine

**Table 4:** Multivariate associations of initial biomarker levels with demographic characteristics and comorbidities^a^

	Arginine		ADMA		SDMA		Arginine/ADMA ratio	
	Coefficient (95% CI)	*p* value	Coefficient (95% CI)	*p* value	Coefficient (95% CI)	*p* value	Coefficient (95% CI)	*p* value
Patient characteristics								
Age in decades	0.01 (−0.02 to 0.03)	0.490	0.03 (0.02 to 0.05)	**<0.001**	0.04 (0.01 to 0.07)	**0.012**	−0.02 (−0.05 to −0.002)	**0.035**
Female	0.0001 (−0.08 to 0.08)	0.997	0.02 (−0.04 to 0.07)	0.506	0.02 (−0.07 to 0.12)	0.639	−0.04 (−0.11 to 0.03)	0.277
BMI	−0.01 (−0.01 to 0.0004)	0.066	0.003 (−0.002 to 0.01)	0.300	0.00 (−0.01 to 0.01)	0.819	−0.01 (−0.01 to −0.003)	**0.005**
Systolic BP in 10 mmHg	0.01 (−0.0003 to 0.03)	0.118	0.001 (−0.01 to 0.01)	0.847	−0.01 (−0.04 to 0.01)	0.195	0.01 (−0.004 to 0.03)	0.154
Comorbidities								
Coronary artery disease	−0.08 (−0.18 to 0.03)	0.160	−0.05 (−0.12 to 0.02)	0.142	−0.02 (−0.15 to 0.10)	0.703	−0.02 (−0.11 to 0.07)	0.605
Congestive heart failure	−0.02 (−0.14 to 0.10)	0.723	0.02 (−0.06 to 0.10)	0.641	0.10 (−0.05 to 0.24)	0.194	−0.06 (−0.17 to 0.04)	0.213
Cerebrovascular disease	0.02 (−0.13 to 0.17)	0.776	−0.02 (−0.12 to 0.08)	0.688	−0.003 (−0.19 to 0.18)	0.970	0.02 (−0.1 to 0.15)	0.726
PAOD	−0.02 (−0.21 to 0.16)	0.814	−0.10 (−0.22 to 0.02)	0.101	0.03 (−0.19 to 0.25)	0.771	0.06 (−0.09 to 0.21)	0.415
Chronic renal failure	0.02 (−0.08 to 0.12)	0.659	0.01 (−0.06 to 0.08)	0.807	0.22 (0.09 to 0.34)	**0.001**	−0.00008 (−0.09 to 0.09)	0.999
Diabetes mellitus	0.02 (−0.09 to 0.13)	0.701	−0.004 (−0.08 to 0.07)	0.923	0.06 (−0.08 to 0.20)	0.406	−0.03 (−0.13 to 0.07)	0.596
Neoplastic disease	0.07 (−0.05 to 0.19)	0.274	0.09 (0.01 to 0.17)	**0.024**	0.11 (−0.04 to 0.25)	0.144	−0.06 (−0.16 to 0.04)	0.273
COPD	−0.02 (−0.11 to 0.06)	0.593	−0.06 (−0.12 to 0.001)	0.056	0.11 (0.001 to 0.22)	**0.047**	0.04 (−0.04 to 0.11)	0.345

Data for multivariate linear regression analyses are given as coefficient (95% CI)

Bolded *p* values are statistically significant at *p* < 0.05

*Abbreviations*: *ADMA* asymmetric dimethylarginine, *BMI* body mass index, *CI* confidence interval, *COPD* chronic obstructive pulmonary disease, *PAOD* peripheral artery occlusive disease, *SDMA* symmetric dimethylarginine

^a^Multivariate model: adjusted for patient age and comorbidities (coronary artery disease, congestive heart failure, cerebrovascular disease, PAOD, chronic renal failure, diabetes mellitus, neoplastic disease, COPD)

## Discussion

The findings of this first study to investigate arginine, ADMA, and SDMA levels in CAP patients revealed that high levels of ADMA and SDMA measured upon ED admission are strongly associated with mid- and long-term mortality, and that high levels of SDMA are associated with short-term mortality. However, this effect wore off after adjustment for comorbidities and age. That leads us to the assumption that this association is mainly defined by the correlation of the biomarkers to several comorbidities like chronic renal failure or COPD and age. Supporting this assumption is the finding that SDMA is also well correlated with PSI but not with the qSOFA score. In contrast, the other biomarkers were not correlated with any of these scores. Arginine did not show any association with mortality or any secondary outcome.

High levels of SDMA and ADMA are indeed associated with mortality [10, 17–19], but we found that this correlation is mainly defined by comorbidities and age. Several studies have found ADMA and SDMA to be associated with chronic renal failure, COPD, asthma, sepsis, cardiovascular risk factors, and cardiovascular and all-cause mortality [2, 3, 6, 10–17]. In short, these two biomarkers are associated with diseases characterized by vascular and bronchial dysfunction. This association could be causal or caused by an imbalance of nitric oxide production [2, 3]. That does not diminish the prognostic value of these markers, but instead shows that they rather provide an overview of the general health status of a patient. This strong correlation with comorbidities, particularly with renal failure, partly explains the good association with the pneumonia risk scores. Our study transfers these findings to patients with CAP, where an association of ADMA, SDMA, and all-cause mortality was found. In contrast to O’Dwyer et al. we did not find a correlation of these biomarkers with sepsis severity [16]. One reason may be, that we assessed sepsis severity by the qSOFA score and O’Dwyer by the SOFA score, which has more power. The other reason may be the limitation, that we did not assess the qSOFA in the initial trial. So we have to consider that we underestimate the score because of the lack of relevant information in some patients (see below in the limitations).

Two earlier studies found that a lower ratio of arginine to ADMA is a more sensitive risk marker for vascular dysfunction and mortality in shock patients than ADMA alone [21, 22]. In our population, we did not find any advantage of the ratios due to the lack of correlation of arginine with mortality and other outcomes.

Hypertension results in higher levels of ADMA and SDMA [6, 10]. It has also been seen that administration of ADMA results in elevated blood pressure in healthy humans [13]. This suggests that antagonising ADMA or SDMA is a potential new target in the therapy of hypertension or renal failure. One way to reach this goal is L-arginine supplementation. In animal studies, long-term L-arginine supplementation seemed to be safe [35, 36]. In rats it showed an attenuation of angiotensin-II - mediated vasoconstriction [37] but in mice with type 1 diabetes it showed no prevention or reduction of renal injury [38].

Raising arginine levels in critically ill human patients by L-arginine supplementation has shown controversial results relating to safety and benefit [39–43]. A meta-analysis of double-blinded, randomized controlled trials showed a significant lowering of systolic and diastolic blood pressure following supplementation of L-arginine [44]. However, there is still little evidence for supplementation in patients with hypertension and there are no studies that investigate the development of complications and progression of chronic renal failure after administration of L-arginine.

In summary, and in contrast to previous studies, our analysis did not reveal ADMA and SDMA to be independent risk factors for all-cause mortality in CAP patients [10, 17]. However, in agreement with earlier investigations, our study showed that SDMA levels detected herein were strongly and independently associated with renal failure, closely related to the glomerular filtration rate [6], and associated with COPD [2, 15], and that ADMA and SDMA are associated with age. The independent association with age may be due partly to the association of ADMA and SDMA with subclinical atherosclerosis [45, 46].

The well-defined cohort of patients with CAP of different severities is one of the main strengths of this study. It is representative of patients usually treated in hospital EDs. The long follow up over a period of 6 years, the high rate of all-cause mortality, and the highly precise laboratory measurement methods are also worth mentioning. A limitation of our study was that it was carried out in hospitals in Switzerland and the results may not be generalizable to other countries. In addition, autopsies were not performed to validate the cause of death, because of which this study only focused on all-cause mortality. Third, this was only a hypothesis-generating observational study. Fourth, the qSOFA score was not measured at ED admission, as a result of which we had to calculate the score on the basis of the information we had. We took data on confusion from the PSI score and counted it as an altered state of mind. Also not all of our patients had information about the respiratory rate and the others had only an estimated rate. We therefore have to assume that we undervalued the general level of the qSOFA score. We also did not have data on the severity of comorbidities (e.g., we only knew that patients had a chronic kidney disease and not the stage of the kidney disease). We also did not measure NO levels and so cannot be sure that our conclusions are complete. Finally, we did not have a complete marker set available for all patients due to low blood specimen volume. Nevertheless, our hypothesis-generating study provides valuable information that will facilitate the design and conduct of additional studies in this area.

## Conclusion

Deducing from our findings, we may assume that neither SDMA nor ADMA nor L-arginine are independently associated with all-cause mortality in CAP patients, although strong unadjusted associations of SDMA—more than ADMA—with mortality and comorbidities are seen in these patients. It remains unclear if higher ADMA and SDMA levels cause higher mortality or if higher mortality causes high levels of ADMA and SDMA, and the precise role NO plays in this scheme of events.

Given our findings, future studies should focus on the causal connection between ADMA and SDMA levels and mortality and on investigating the clinical benefit of antagonising ADMA and SDMA (for example, with L-arginine).

## Abbreviations

ADMA: Asymmetric dimethylarginine
AUC: Area under the receiver operating characteristic curve
BMI: Body mass index
CAD: Coronary artery disease
CAP: Community-acquired pneumonia
CHF: Congestive heart failure
CI: Confidence interval
CRF: Chronic renal failure
CRP: C-reactive protein
DDAH: Dimethylarginine dimethylaminohydrolase
DM: Diabetes mellitus
ED: Emergency department
HAP: Hospital-acquired pneumonia
HR: Hazard ratio
IQR: Interquartile range
LC-MS/MS: Liquid chromatography tandem-mass spectrometry
NO: Nitric oxide
NOS: Nitric oxide synthase
PCT: Procalcitonin
PRMT: Protein arginine methyl transferase
PSI: Pneumonia severity index
qSOFA: Quick Sepsis related Organ Failure Assessment: altered mental status, fast respiratory rate (≥22 breaths per min), low blood pressure (systolic blood pressure ≤100 mmHg)
SDMA: Symmetric dimethylarginine
